# Editorial: The Effect of the COVID-19 Pandemic on Cancer Patients and Healthcare

**DOI:** 10.3389/fonc.2022.859903

**Published:** 2022-03-03

**Authors:** Nicola Silvestris, Oronzo Brunetti, Antonio Galvano, Antonio Russo, Giovanni Apolone

**Affiliations:** ^1^ Medical Oncology Unit, Department of Human Pathology “G. Barresi”, University of Messina, Messina, Italy; ^2^ Medical Oncology Unit, IRCCS Istituto Tumori “Giovanni Paolo II”, Bari, Italy; ^3^ Department of Surgical, Oncological and Stomatological Sciences, University of Palermo, Palermo, Italy; ^4^ Scientific Direction - Fondazione IRCCS Istituto Nazionale dei Tumori di Milano, Milan, Italy

**Keywords:** COVID-19, SARS-CoV-2, immunocompromised patient, Cancer, healthcare (MeSH)

Coronavirus disease 2019 (COVID-19), caused by the severe acute respiratory syndrome coronavirus 2 (SARS-CoV-2), has been classified as a global pandemic (1). Preliminary evidence suggests that the incidence of severe COVID-19 is increased with increasing age and the presence of underlying conditions such as hypertension or diabetes (1). The progression of viral infections in immunocompromised patients is also high, and an immunocompromised state associated with cancer or transplantation is considered a risk factor for developing severe COVID-19 conditions (2, 3). Although SARS-CoV-2 infection in cancer patients can make management a challenge and make a burden on the patients and government, there are still many controversies about which SARS-CoV-2 infection can make cancer worse or not. However, researchers highlighted that COVID-19 has a particularly negative effect on patients with cancer (4). With the onset of COVID-19, the medical community is faced with several issues, including increased competition for healthcare resources. The oncology community had an immediate requirement to protect patients thought vulnerable to a possibly lethal infection. The impact of the pandemic on clinically significant alterations in either primary, due to changes in timing or therapy method, or secondary, due to changing combined effects on disease development or therapeutic outcomes (Izadmehr et al.). Still, cancer therapy in patients with SARS-CoV-2 infection has been the subject of much debate in recent years. Two studies from China suggested that chemotherapy was associated with worse results.

In contrast, published larger trials found that administration of cytotoxic drugs within four weeks of a positive SARS-CoV-2 test had no significant effect on infection-associated adverse outcomes and patient mortality (Gurizzan et al.). A study concerning the impact of COVID-19 on clinical characteristics in patients with lung cancer showed that the COVID-19 outbreak surprisingly had a positive effect on early-stage lung cancer screening. One hundred ten patients containing 47.6 percent of all new lung cancer cases were detected by COVID-19 screening, which made early detection and treatment (Zhang et al.). The other study conducted by Al-Mozaini et al. has proved the fact that cancer patients with a high viral load had an in-hospital death rate of 41.38 percent, compared to 23.81 percent for those with a medium viral burden and 14.29 percent for those with a low viral load (*p* < 0.01). Also, they highlighted that a high viral load was linked to an increased risk of severe outcomes in patients with lung and hematologic cancers. In cancer patients undergoing anticancer treatment, higher viral loads were associated with an increased risk of death and morbidity.

Besides the challenges and findings mentioned previously, clinicians must consider COVID-19 as a complex disease with a new concept, “syndemic,” as Horton stated (5). The term “syndemic” refers to two or more epidemics cooperating synergistically and contributing to the clustering of an excess burden of disease in an area or population, rather than simply the sum of both. The SARS-CoV-2 epidemic overlaps with endemic diseases such as malaria, schistosomiasis, tuberculosis, hepatitis C, and seasonal illnesses like influenza and other respiratory infections, affecting cultural and social determinants. SARS-CoV-2 has elucidated the complex dynamics between a new, complicated health problem, coexisting, chronic, and endemic health problems. A syndemic perspective on health and disease is required for this and future epidemics (6). So, a more sophisticated strategy is required to maintain our communities’ health due to the syndemic nature of the challenge we are facing.

## Author Contributions

NS: conceptualization and supervision. OB: writing. AG, AR, and GA: writing and editing. All authors contributed to the article and approved the submitted version.

## Conflict of Interest

The authors declare that the research was conducted in the absence of any commercial or financial relationships that could be construed as a potential conflict of interest.

## Publisher’s Note

All claims expressed in this article are solely those of the authors and do not necessarily represent those of their affiliated organizations, or those of the publisher, the editors and the reviewers. Any product that may be evaluated in this article, or claim that may be made by its manufacturer, is not guaranteed or endorsed by the publisher.
